# Study of Intrinsic Dissipation Due to Thermoelastic Coupling in Gyroscope Resonators

**DOI:** 10.3390/s16091445

**Published:** 2016-09-07

**Authors:** Changlong Li, Shiqiao Gao, Shaohua Niu, Haipeng Liu

**Affiliations:** State Key Laboratory of Explosion Science and Technology, Beijing Institute of Technology, Beijing 100081, China; xiaolong_joy@163.com (C.L.); gaoshq@bit.edu.cn (S.G.); lhp@bit.edu.cn (H.L.)

**Keywords:** gyroscope resonator, intrinsic dissipation, thermoelastic damping, quality factor, drive-mode, sense-mode

## Abstract

This paper presents analytical models, as well as numerical and experimental verification of intrinsic dissipation due to thermoelastic loss in tuning-fork resonator. The thermoelastic analytical governing equations are created for resonator vibrating at drive-mode and sense-mode, and thermoelastic vibration field quantities are deduced. Moreover, the theoretical values are verified that coincided well with finite element analysis (FEM) simulation results. Also, the comparison of vibration field quantities is made to investigate the effect of different conditions on resonator thermoelastic vibration behavior. The significant parameters of thermoelastic damping and quality factor are subsequently deduced to analyze the energy dissipation situation in the vibration process. Meanwhile, the corresponding conclusions from other studies are used to verify our theoretical model and numerical results. By comparing with the experimental quality factor, the numerical values are validated. The combination of the theoretical expressions, numerical results and experimental data leads to an important insight into the achievable quality factor value of tuning-fork resonator, namely, that the thermoelastic damping is the main loss mechanism in the micro-comb finger structure and the quality factor varies under different vibration modes. The results demonstrate that the critical geometry dimensions of tuning-fork resonator can be well designed with the assistance of this study.

## 1. Introduction

Micro-resonators as critical components in microelectromechanical systems (MEMS), such as accelerometers, gyroscopes, harvesters etc., have considerably aroused the interest of researchers [1,2,3]. For all these applications, the prevalent parameter that has emerged is the resonator’s quality factor (*Q*) which is described as mechanical energy damping. In the MEMS field, it is important to design and fabricate micro-resonators with very high quality factors or very little energy loss, because a high quality factor results in high sensitivity and low power consumption [4]. Energy dissipation can occur in micro-resonators through intrinsic and external energy loss. The thermoelastic damping (TED) and the lattice defects belong to intrinsic loss while the air damping belongs to external energy loss. TED has been identified as the fundamental limit for attainable high quality factor of micro-resonators.

TED results from the irreversible heat flow in the vibration process of resonators. When a mechanical resonator vibrates, compressive stress occurs in some regions while tensile stress affects other regions. Accordingly, compressed regions heat up while stretched regions cool down. Hence a temperature gradient is established between different regions of a system. Thus, the system will adjust itself to the thermal equilibrium state by thermal conduction. However, the energy used in the adjustment process cannot be restored after the system returns to its original state. As long as the structure’s thermal expansion coefficient is not zero, TED will exist in micro-resonators. In recent years, much work has been done on the TED of micro-resonators. Zener derived the analytical model of TED and predicted thermoelastic losses which may be a limitation to the maximum Q of resonators [5,6]. Lifshitz and Rouckes gave the derivation of an exact expression for thermoelastic damping in thin rectangular beams and compared it with Zener’s approximation [7]. Rezazadeh et al. studied TED in a microbeam with modified couple stress theory [8]. Jiao et al. presented the numerical results of thermoelastic damping [9,10]. Kausinis used COMSOL Multiphysics to calculate damping in micromechanical resonator structures [11]. Guo used a customized finite element method to evaluate the thermoelastic damping in micro-beam resonators [12]. Yi used a reduced finite element formulation to investigate the thermoelastic damping in contour-mode in-plane vibrations [13].

In the field of MEMS applications, as different resonators are used, many researchers directtheir attention to the TED for various resonators. Lifshitz and Rouckes researched the TED effect in thin rectangle beam. Amy Duwel and X. Guo presented the TED-based methodology to calculate the microbeam resonator with different boundary conditions [14]. Nayfehprovided a model and analytical expressions for the quality factors of microplates with TED [15]. De studied the TED of electrostatically actuated resonators and Abouelregal researched the TED of an axially moving microbeam with external loading [16,17]. Bassiouny presented a model for a layered thin plate of sandwich structure, which is studied with the theory of TED [18].

Although a tremendous amount of work has been done on thermoelastic coupling of micro-resonators in MEMS, very little research has been systematically undertaken on the gyroscope resonator with different vibration states. Weijian has researched the longitudinal vibration of the micro-resonators and made a comparison with the case of flexural vibration [9], and Sharmahas studied the thermoelastic damping of 3-D cantilever beam [19]. Therefore, the gyroscope has two vibration manners: the longitudinal vibration and the transverse vibration with Coriolis Effect. Thus, for 3D SOI-based resonators, both the longitudinal vibration and transverse vibration should be considered when analyzing TED of MEMS gyroscope. Also, the thermoelastic behavior of micro-resonators for free and forced vibration with electrostatic load should be taken into account. 

On the basis of previous work, this paper presents the tuning-fork resonator model that illustrates the thermoelastic coupling behavior. The micro-comb fingers model is established for analyzing the thermoelastic coupling effect both in free vibration and electrostatic-actuated vibration. The governing equations of coupled thermoelastic problems for two different vibration conditions are set up, before the equations to analyze the influence of TED are calculated. The field quantities of displacement and temperature distribution are analyzed and the TED factor is calculated under different conditions. The corresponding finite element analysis (FEM) is made to verify the numerical results. Finally, the significant parameter of Q is expressed as the reciprocal of TED, compared with the experimental conclusion which is reported in the literature.

## 2. Theoretical Description of Thermoelastic Coupling Effects in Micro-Resonators

### 2.1. The Micro-Resonator Model Design 

**Figure 1:**
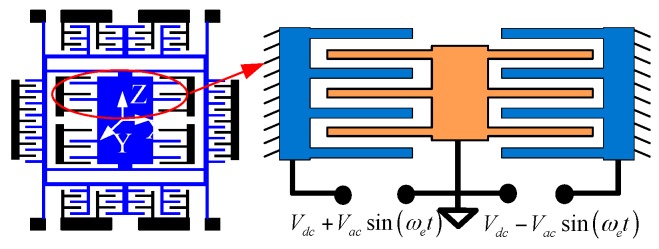
Schematic view of an electrostatic comb structure.

The critical component of gyroscope is the differential comb fingers resonator which acts as the driven element. When voltage is applied to the resonator, the electrostatic force will be generated correspondingly. The electrical load is composed of two components, which is AC and DC voltage. The applied DC voltage deforms the upper elastic surface that causes change in the system capacitance. If AC voltage is added to the DC one, the resonator that has harmonic motion can be obtained. Figure 1 shows a schematic view of a tuning-fork resonator which is consisted of a set of the movable and fixed fingers. As illustrated in Figure 1, the voltage that applied in the fixed comb is $V_{dc}+V_{ac}\sin\left(\omega_{e}t\right)$ and $V_{dc}-V_{ac}\sin\left(\omega_{e}t\right)$, respectively. 

The system capacitance change value is

(1) $$\Delta C=\frac{2\varepsilon bx}{g}$$

where ε is dielectric constant; *b* and *g* are thickness of comb and gap between the combs respectively.

Then, the electrostatic force can be presented as follows.

(2) $$F_{e}=\frac{1}{2}\frac{\partial C}{\partial x}V^{2}=N\varepsilon \frac{b}{g}V_{dc}V_{ac}\sin(\omega_{e}t)$$

Among it, $V_{dc}$ represents direct voltage; $V_{ac}$ refers to altering voltage; $\omega_{e}$ stands for excitation frequency, *N* represents the number of comb fingers.

**Figure 2:**
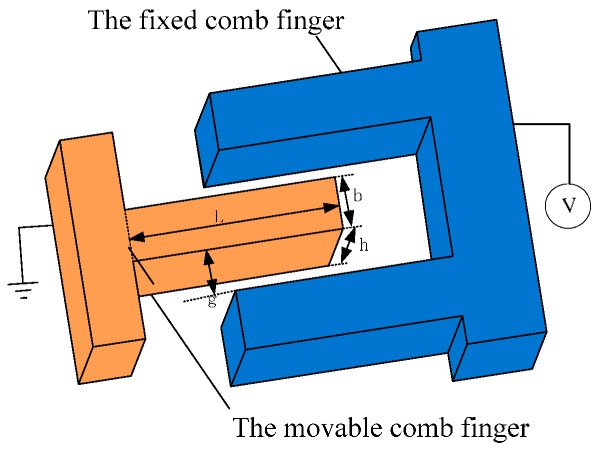
Illustration of a group of comb finger.

To investigate the effect of TED on tuning-fork resonator, a group of comb fingers is selected as the research object. Figure 2 illustrates the model schematic. From the model schematic view, the movable finger is modeled with dimensions, 0<x<L, −b/2<y<b/2 and −h/2<z<h/2. We define the x, y, z axes corresponding to the length, width and thickness, respectively. In the equilibrium, the initial temperature of resonator is $T_{0}$ everywhere.

### 2.2. Governing Equations of Resonator under Electrostatic-Forced Vibration and Free Vibration

In the longitudinal vibrating process, each cross-section of the micro-structure remains plane and the transverse deformation is ignored. For the longitudinal vibration problem, all quantities are only dependent on variable *x*. *u(x, t)* is defined as a longitudinal deflection of the beam. The thermoelastic governing equation of the electrostatic load is written as Equation (3):

(3) $$\left\{ \begin{array}{l} \rho \frac{\partial^{2}u}{\partial t^{2}}-E\frac{\partial^{2}u}{\partial x^{2}}+\frac{\alpha E}{1-2\upsilon }\frac{\partial \theta }{\partial x}=F\\ \kappa \frac{\partial^{2}\theta }{\partial x^{2}}-\rho C_{v}\frac{\partial \theta }{\partial t}-\alpha ET_{0}\frac{\partial^{2}u}{\partial x\partial t}=0 \end{array}\right.$$

where ρ, E, α, F, κ, $C_{v}$, t and υ are density, Young’s modulus, thermal expansion coefficient, electrostatic force per unit volume, thermal conductivity, specific heat capacity, time and Poisson’s ratio, respectively. Also, $\theta =T-T_{0}$ is temperature variation in which T is defined as temperature field of the resonator. The first equation is deduced from the vibration equilibrium equation and the second equation is derived from the thermal dynamic equation.

In order to compare the thermoelastic coupling effect on various vibration forms of resonator, the free harmonic vibration thermoelastic coupling equation is established as follows.

(4) $$\left\{ \begin{array}{l} \rho \frac{\partial^{2}u}{\partial t^{2}}-E\frac{\partial^{2}u}{\partial x^{2}}+\frac{\alpha E}{1-2\upsilon }\frac{\partial \theta }{\partial x}=0\\ \kappa \frac{\partial^{2}\theta }{\partial x^{2}}-\rho C_{v}\frac{\partial \theta }{\partial t}-\alpha ET_{0}\frac{\partial^{2}u}{\partial x\partial t}=0 \end{array}\right.$$

As the gyroscope vibrates at the driven model, the micro-structure performs longitudinal vibration. Then with the Coriolis Effect, the resonator transverse vibrates at the detection mode. Accordingly, the flexural vibration governing the movable finger equation within the thermoelastic coupling effect is given by the following equation.

(5) $$\left\{ \begin{array}{l} \rho A\frac{\partial^{2}w}{\partial t^{2}}+EI\frac{\partial^{4}w}{\partial x^{4}}+\frac{\partial^{2}M_{T}}{\partial x^{2}}=0\\ {\left(\frac{E\alpha }{1-2\upsilon }\right)}^{2}IT_{0}\frac{\partial }{\partial t}\frac{\partial^{2}w}{\partial x^{2}}+\kappa \frac{\partial^{2}M_{T}}{\partial x^{2}}-\kappa p^{2}M_{T}-\rho C_{v}\frac{\partial M_{T}}{\partial t}=0 \end{array}\right.$$

As is noted, the cross-section of the micro-resonator is rectangular, the area and moment of inertia of the cross-section are A=bh and $I=bh^{3}/12$ where h is the thickness. $M_{T}=\frac{E\alpha b}{1-2\upsilon }\int_{-h/2}^{h/2}\theta zdz$ is thermal moment and w(x, t)refers to transverse deflection of the resonator. In Equation (5), *p* is equal to π/h. 

## 3. Analysis Methods of the Coupling Equations

To evaluate the coupling strength between structure mechanical and temperature fields, the analytical approaches are used to solve three governing equations. Therefore, the direct coupling method can be used to solve the thermoelastic coupling problems in micro-comb fingers resonator with and without electrostatic load in drive-mode of longitudinal vibration and in sense-mode of transverse vibration. 

### 3.1. Direct Coupling Method in Resonator with Electrostatic Load

For the sake of solving Equation (3), the following dimensionless variables are used to transform Equation (3) into a non-dimensional form.

(6) $$\bar{u}=\frac{u}{L},\bar{x}=\frac{x}{L},\;\bar{\theta }=\frac{\theta }{T_{0}},\;\bar{t}=\frac{t}{t_{0}},\;t_{0}=L/\sqrt{E/\rho },\bar{F}=\frac{F}{EA}$$

Therefore, the governing equation in its non-dimensional form is simplified as:

(7) $$\left\{ \begin{array}{l} \frac{\partial^{2}\bar{u}}{\partial {\bar{t}}^{2}}-\frac{\partial^{2}\bar{u}}{\partial {\bar{x}}^{2}}+a_{1}\frac{\partial \bar{\theta }}{\partial \bar{x}}=\bar{F}\\ \frac{\partial^{2}\bar{\theta }}{\partial {\bar{x}}^{2}}-a_{2}\frac{\partial \bar{\theta }}{\partial \bar{t}}-a_{3}\frac{\partial^{2}\bar{u}}{\partial \bar{x}\partial \bar{t}}=0 \end{array}\right.$$

where $a_{1}=\frac{\alpha T_{0}}{1-2\upsilon }$, $a_{2}=\frac{\rho C_{v}L^{2}}{\kappa t_{0}}$, $a_{3}=\frac{\alpha EL^{2}}{\kappa t_{0}}$.

In the interest of dealing with the problem, both initial and boundary conditions should be taken into consideration. The initial conditions of temperature and displacement are assumed to be homogeneous. The initial conditions and boundary conditions are presented as below.

(8) $$\left\{ \begin{array}{l} u{|}_{t=0}=\frac{\partial u}{\partial t}{|}_{t=0}=0,\;\theta {|}_{t=0}=\frac{\partial \theta }{\partial t}{|}_{t=0}=0\\ \frac{\partial u}{\partial x}{|}_{x=0,L}=0,\;{\theta |}_{x=0}=\frac{\partial \theta }{\partial x}{|}_{x=L}=0 \end{array}\right.$$

The same transformation is used for the initial and boundary conditions. Equation (8) is rewritten as follows.

(9) $$\left\{ \begin{array}{l} \bar{u}{|}_{\bar{t}=0}=\frac{\partial \bar{u}}{\partial \bar{t}}{|}_{\bar{t}=0}=0,\;\bar{\theta }{|}_{\bar{t}=0}=\frac{\partial \bar{\theta }}{\partial \bar{t}}{|}_{\bar{t}=0}=0\\ \frac{\partial \bar{u}}{\partial \bar{x}}{|}_{\bar{x}=0,1}=0,\;{\bar{\theta }|}_{\bar{x}=0}=\frac{\partial \bar{\theta }}{\partial \bar{x}}{|}_{\bar{x}=1}=0 \end{array}\right.$$

To solve the mechanical and thermal coupled Equation (3) more conveniently, the Laplace transform is introduced. The Laplace transform is defined as:

(10) $$X(s)=\int_{0}^{\infty }x(t)e^{-st}dt$$

Substituting Equation (10) into Equation (3), the result is

(11) $$\left\{ \begin{array}{l} s^{3}U-{\frac{d\bar{u}}{d\bar{t}}|}_{\bar{t}=0}-s^{2}{u|}_{\bar{t}=0}-\frac{d^{2}U}{d{\bar{x}}^{2}}-a_{1}\frac{d\Theta }{d\bar{x}}=a_{2}\bar{F}\\ \frac{d^{2}\Theta }{d{\bar{x}}^{2}}-a_{2}s^{2}\Theta +a_{2}s{\bar{\theta }|}_{\bar{t}=0}-a_{3}s^{2}\frac{dU}{d\bar{x}}+a_{3}s{\bar{u}|}_{\bar{t}=0}=0 \end{array}\right.$$

The initial equations are substituted into Equation (11), which can be rewritten as below.

(12) $$\left\{ \begin{array}{l} s^{3}U-\frac{d^{2}U}{d{\bar{x}}^{2}}+a_{1}\frac{d\Theta }{d\bar{x}}=a_{2}\bar{F_{s}}\\ \frac{d^{2}\Theta }{d{\bar{x}}^{2}}-a_{2}s^{2}\Theta -a_{3}s^{2}\frac{dU}{d\bar{x}}=0 \end{array}\right.$$

U, Θ and $\bar{F_{s}}$ denote themselves Laplace transforms, respectively, and s denotes the Laplace transform parameter. The differential equations for U and Θ are given by using the elimination methodin Equation (12).

(13) $$\left\{ \begin{array}{l} (\frac{d^{4}}{d{\bar{x}}^{4}}+A_{1}\frac{d^{2}}{d{\bar{x}}^{2}}+A_{2})U=A_{3}\bar{F_{s}}\\ (\frac{d^{4}}{d{\bar{x}}^{4}}+A_{1}\frac{d^{2}}{d{\bar{x}}^{2}}+A_{2})\Theta =0 \end{array}\right.$$

where $A_{1}=-a_{1}a_{3}s^{2}-a_{2}s^{2}-s^{3}$, $A_{2}=a_{2}s^{5}$, $A_{3}=a_{2}s^{2}$

The solution to Equation (13) in the Laplace domain can be presented as:

(14) $$\left\{ \begin{array}{l} U=\frac{A_{3}}{A_{2}}\bar{F_{s}}+\sum_{i=1}^{4}C_{i}e^{m_{i}x}\\ \Theta =\sum_{i=1}^{4}C_{i}^{\prime }e^{m_{i}x} \end{array}\right.$$

where $C_{i}$ and $C_{i}^{\prime }$ are parameters depending on *s* and $m_{i},i=1,2,3,4$ are the roots of the characteristic equation: $m^{4}+A_{1}m^{2}+A_{2}=0$.

Substituting Equation (14) into differential equation about Θ gives the compatibility between $C_{i}$ and $C_{i}^{\prime }$.$C_{i}^{\prime }=\beta_{i}C_{i}$, where $\beta_{i}=\frac{a_{3}s^{2}m_{i}}{m_{i}^{2}-a_{2}s}$.

Governing Equation (14) can be represented as:

(15) $$\left\{ \begin{array}{l} U=\frac{A_{3}}{A_{2}}\bar{F_{e}}+\sum_{i=1}^{4}C_{i}e^{m_{i}x}\\ \Theta =\sum_{i=1}^{4}\beta_{i}C_{i}e^{m_{i}x} \end{array}\right.$$

Then, the boundary conditions of Laplace transform to Equation (9) are

(16) $${\frac{\partial U}{\partial \bar{x}}|}_{\bar{x}=0,1}=0,\;{\Theta_{\bar{x}=0}=\frac{\partial \Theta }{\partial \bar{x}}|}_{\bar{x}=1}=0$$

Substituting Equation (15) into the above boundary Equation (16), the four linear equations in the matrix form can be obtained as follows.

(17) $$\left[\begin{array}{cccc} m_{1} & m_{2} & m_{3} & m_{4}\\ m_{1}e^{m_{1}} & m_{2}e^{m_{2}} & m_{3}e^{m_{3}} & m_{4}e^{m_{4}}\\ \beta_{1} & \beta_{2} & \beta_{3} & \beta_{4}\\ \beta_{1}m_{1}e^{m_{1}} & \beta_{2}m_{2}e^{m_{2}} & \beta_{3}m_{3}e^{m_{3}} & \beta_{4}m_{4}e^{m_{4}} \end{array}\right]\left[\begin{array}{l} C_{1}\\ C_{2}\\ C_{3}\\ C_{4} \end{array}\right]=\left[\begin{array}{l} 0\\ 0\\ 0\\ 0 \end{array}\right]$$

$C_{i}$ of the solution of the above linear equations is the unknown parameters. It is difficult to find the inverse Laplace transform of temperature and displacement distribution in the Laplace domain analytically. In order to determine the temperature variation and displacement distributions in the time domain, the Riemann-sum approximation method is used to obtain the numerical results [20]. In this method, any function in the Laplace domain can be inverted to that in the time domain. The Riemann-sum approximation method is defined as below.

(18) f(t)=eζtt[12Re[F¯(ζ)]+Re∑n=0NF¯(ζ+inπt)(−1)n]

where Re and i are real part and imaginary part, respectively. For fast convergence, numerical experiments have shown that ζ equals to 4.7/t, which can meet the above relation.

### 3.2. Direct Coupling Method in Resonator without Electrostatic Load

The Laplace transform is used in Equation (4) for comparing thermoelastic behavior in the micro-resonator with electrostatic-forced vibration before the dimensionless governing equation in free harmonic vibration is listed as.

(19) $$\left\{ \begin{array}{l} (\frac{d^{4}}{d{\bar{x}}^{4}}+A_{1}\frac{d^{2}}{d{\bar{x}}^{2}}+A_{2})U^{\prime }=0\\ (\frac{d^{4}}{d{\bar{x}}^{4}}+A_{1}\frac{d^{2}}{d{\bar{x}}^{2}}+A_{2})\Theta^{\prime }=0 \end{array}\right.$$

where $A_{1}$, $A_{2}$ are defined as in Equation (13). The solution to Equation (19) can be obtained as follows.

(20) $$\left\{ \begin{array}{l} U^{\prime }=\sum_{i=1}^{4}G_{i}e^{m_{i}x}\\ \Theta^{\prime }=\sum_{i=1}^{4}G_{i}^{\prime }e^{m_{i}x} \end{array}\right.$$

Also, $G_{i}^{\prime }=\beta_{i}G_{i}$ where $\beta_{i}=a_{3}s^{2}m_{i}/\left(m_{i}^{2}-a_{2}s\right)$. Applying the same boundary condition in Equation (8) to Equation (20), another four linear equations in the matrix form can be obtained as follows.

(21) $$\left[\begin{array}{cccc} m_{1} & m_{2} & m_{3} & m_{4}\\ m_{1}e^{m_{1}} & m_{2}e^{m_{2}} & m_{3}e^{m_{3}} & m_{4}e^{m_{4}}\\ \beta_{1} & \beta_{2} & \beta_{3} & \beta_{4}\\ \beta_{1}m_{1}e^{m_{1}} & \beta_{2}m_{2}e^{m_{2}} & \beta_{3}m_{3}e^{m_{3}} & \beta_{4}m_{4}e^{m_{4}} \end{array}\right]\left[\begin{array}{l} G_{1}\\ G_{2}\\ G_{3}\\ G_{4} \end{array}\right]=\left[\begin{array}{l} 0\\ 0\\ 0\\ 0 \end{array}\right]$$

### 3.3. Analytical Approaches to Resonator in Transverse Vibration

In gyroscope sense-mode, the movable comb finger vibrates in flexible vibration. In the resonator, the Euler-Bernoulli assumption is used so that any plane cross-section initially is perpendicular to the axis of the beam and remains plane during bending.

To solve the coupling Equation (5), the assumption is made that the thermal moment $M_{T}$ and displacement component w are time-harmonic forms.

(22) $$w(x,t)=w\left(x\right)e^{i\omega t},\;M_{T}(x,t)=M_{T}(x)e^{i\omega t}$$

Equation (5) can be represented as

(23) $$\left\{ \begin{array}{l} -\rho A\omega^{2}w+EI\frac{\partial^{4}w}{\partial x^{4}}+\frac{\partial^{2}M_{T}}{\partial x^{2}}=0\\ -\kappa \frac{\partial^{2}M_{T}}{\partial x^{2}}+(\kappa p^{2}+i\omega C_{v})M_{T}=i\omega {\left(\frac{E\alpha }{1-2\upsilon }\right)}^{2}IT_{0}\frac{\partial^{2}w}{\partial x^{2}} \end{array}\right.$$

The boundary conditions of the movable comb finger in flexural vibrating are given by the following equation.

(24) $$\left\{ \begin{array}{l} {w|}_{x=0}={\;\frac{\partial w}{\partial x}|}_{x=0}\;=0,\;{\frac{\partial^{2}w}{\partial x^{2}}|}_{x=l}\;={\frac{\partial^{3}w}{\partial x^{3}}|}_{x=l}=0\\ {\frac{\partial M_{T}}{\partial x}|}_{x=0}={M_{T}|}_{x=l}=0\; \end{array}\right.$$

Differentiating the thermal conduction equation of Equation (5) with respect to *x* and substituting the first equation of Equation (5) into it results in

(25) $$A^{'}\frac{\partial^{6}w}{\partial x^{6}}+B\frac{\partial^{4}w}{\partial x^{4}}+C\frac{\partial^{2}w}{\partial x^{2}}+Dw=0$$

where $\begin{array}{l} A^{'}=\kappa EI\;B=-((\kappa p^{2}+i\omega \rho C_{v})EI+i\omega {\left(\frac{E\alpha }{1-2\upsilon }\right)}^{2}IT_{0})\\ C=-\kappa \rho S\omega^{2}\;D=(\kappa p^{2}+i\omega \rho C_{v})\rho S\omega^{2} \end{array}$

The solution of Equation (25) can be expressed as

(26) $$w(x)=\sum_{m=1}^{3}\left(L_{m}\sinh(r_{m}x)+N_{m}\cosh(r_{m}x)\right)$$

where $\pm r_{m}(m=1,2,3)$ are the roots of the equation of $A^{'}r^{6}+Br^{4}+Cr^{2}+D=0$, and $L_{m}$,$N_{m}$ (m=1,2,3) are constants. 

Substituting the dynamic equation of Equation (5) into the thermal conduction equation, the result is as follows.

(27) $$(\kappa p^{2}+i\omega \rho C_{v})M_{T}=\kappa EI\frac{\partial^{4}w}{\partial x^{4}}+i\omega {\left(\frac{E\alpha }{1-2\upsilon }\right)}^{2}IT_{0}\frac{\partial^{2}w}{\partial x^{2}}-\kappa \rho A\omega^{2}w$$

Substituting Equation (26) into Equation (27) leads to

(28) $$M_{T}=\sum_{m=1}^{3}\left(L_{m}h_{m}\sinh(r_{m}x)+N_{m}h_{m}\cosh(r_{m}x)\right)$$

where $h_{m}=\left(-EI\kappa r_{m}^{4}+i\omega {\left(\frac{E\alpha }{1-2\upsilon }\right)}^{2}IT_{0}r_{m}^{2}+\kappa \rho A\omega^{2}\right)/\left(\kappa p^{2}+i\omega \rho C_{v}\right)$

Thus, the solution of displacement component and the temperature moment can be deduced.

(29) $$\left\{ \begin{array}{l} w(x)=\sum_{m=1}^{3}\left(L_{m}\sinh(r_{m}x)+N_{m}\cosh(r_{m}x)\right)\\ M_{T}=\sum_{m=1}^{3}\left(L_{m}h_{m}\sinh(r_{m}x)+N_{m}h_{m}\cosh(r_{m}x)\right) \end{array}\right.$$

Substituting the boundary conditions into Equation (29), Equation (30) can be obtained.

(30) $$\left\{ \begin{array}{l} \sum_{m=1}^{3}\left(N_{m}\right)=0,\;\sum_{m=1}^{3}\left(L_{m}r_{m}\right)=0\\ \sum_{m=1}^{3}\left(L_{m}r_{m}^{2}\sinh(r_{m}l)+N_{m}r_{m}^{2}\cosh(r_{m}l)\right)=0,\;\sum_{m=1}^{3}\left(L_{m}r_{m}^{3}\cosh(r_{m}l)+N_{m}r_{m}^{3}\sinh(r_{m}l)\right)=0\\ \sum_{m=1}^{3}(L_{m}h_{m}r_{m})=0,\;\sum_{m=1}^{3}\left(L_{m}h_{m}\sinh(r_{m}l)+N_{m}h_{m}\cosh(r_{m}l\right))=0 \end{array}\right.$$

The corresponding frequency equations of thermoelastic coupling transverse vibration are:

(31) $$\left|\begin{array}{cccccc} 0 & 1 & 0 & 1 & 0 & 1\\ r_{1} & 0 & r_{2} & 0 & r_{3} & 0\\ r_{1}^{2}\sinh(r_{1}l) & r_{1}^{2}\cosh(r_{1}l) & r_{2}^{2}\sinh(r_{2}l) & r_{2}^{2}\cosh(r_{2}l) & r_{3}^{2}\sinh(r_{3}l) & r_{3}^{2}\cosh(r_{3}l)\\ r_{1}^{3}\cosh(r_{1}l) & r_{1}^{3}\sinh(r_{1}l) & r_{2}^{3}\cosh(r_{2}l) & r_{2}^{3}\sinh(r_{2}l) & r_{3}^{3}\cosh(r_{3}l) & r_{3}^{3}\sinh(r_{3}l)\\ r_{1}h_{1} & 0 & r_{2}h_{2} & 0 & r_{3}h_{3} & 0\\ h_{1}\sinh(r_{1}l) & h_{1}\cosh(r_{1}l) & h_{2}\sinh(r_{2}l) & h_{2}\cosh(r_{2}l) & h_{3}\sinh(r_{3}l) & h_{3}\cosh(r_{3}l) \end{array}\right|\;=0$$

By solving a complex transcendental Equation (31), the frequency ω can be obtained. The numerical method will be employed to solve this equation in the next section.

## 4. Numerical Results

The material used to fabricate the gyroscope is Si, and the parameters in numerical calculation and simulation are listed in Table 1.

### 4.1. Thermoelastic Behaviors of Micro-Comb Finger Vibration with Electrostatic Load

**Figure 3:**
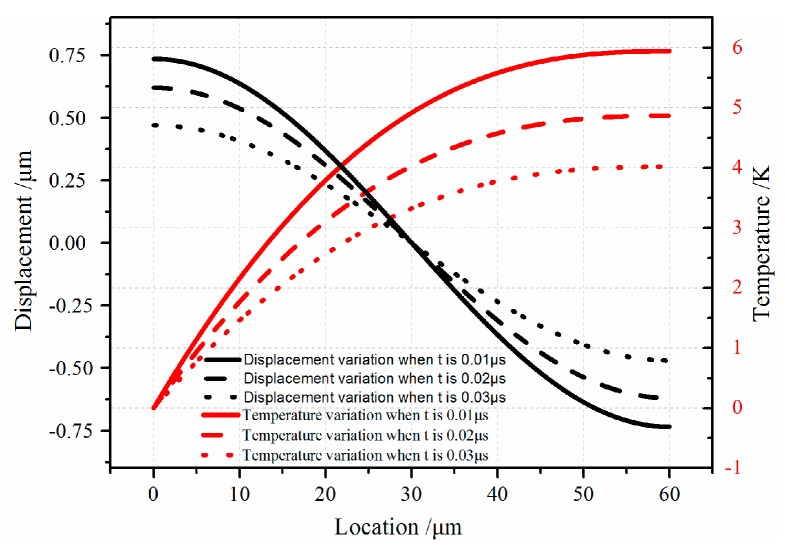
Displacement and temperature variation for different values of vibration width *t* is 0.01 µs, 0.02 µs and 0.03 µs.

The defined Riemann-sum approximation method in Equation (18) is used to perform the numerical inverse Laplace transform. The vibration displacement and temperature that generate during the vibration process are obtained. Figure 3 indicates the dynamic displacement and temperature of the micro-resonator for the coupled thermoelastic case. It can be observed in Figure 3 that the displacement *u* reaches maximum value both in two ends to satisfy the boundary Equation (8). Also, it can be seen from inverse Laplace transform that the vibration of the movable comb finger decays with time increasing when the coupling between strain and thermal fields are taken into account. Also the temperature decreases with the time increasing which means the mechanical energy of resonator is dissipated in the form of thermal energy. From the relationship between vibration displacement and temperature, it can be concluded that the temperature increases with vibration strength. When the resonator vibrates at drive-mode with electrostatic load, the variation of temperature is more severe. 

Comparison is made between the displacement variation for different electrostatic parameters of direct voltage and alternating voltage, respectively. In the calculation process, the non-contrast parameters are kept in initial value (Table 1).

**Figure 4:**
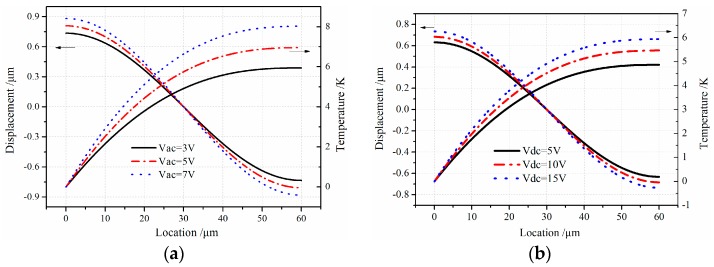
Influence of electrostatic parameters on the field quantities along the axial direction of movable comb finger; (**a**) *u*, *θ* versus Vac; (**b**) *u*, *θ* versus Vdc.

Figure 4 shows the graphical result of field quantities with the variation of the electrostatic parameters for altering voltage and direct voltage. From Figure 4a,b, the field quantities are sensitive to the variation of Vac and Vdc. When micro-comb finger harmonic vibration with AC voltage is applied, the field quantities are more sensitive to Vac. The amplitudes of displacement and temperature variation with the effect of altering voltage are 0.1 µm and 1 K, respectively, and 0.05 µm, 0.5 K with direct voltage. 

### 4.2. Thermoelastic Behaviors of Micro-Comb Finger Vibration without Electrostatic Load

**Figure 5:**
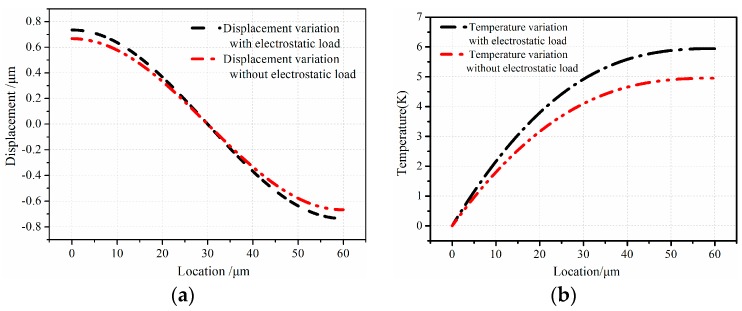
Field quantities variation under different conditions: (**a**) displacement variation with and without electrostatic load; (**b**) temperature variation with and without electrostatic load.

In order to measure the effect of electrostatic load on the thermal and vibration couple process, the vibration with electrostatic load and without load is compared. The results are graphically presented in Figure 5. Note that the longitudinal free vibration moves around the first resonance frequency. From Figure 5, there is a quite obvious difference in the displacement under two different conditions. The discrepancy is about 0.1 µm in displacement, and the temperature variation shows 1.0 K difference. From the results, the thermoelastic coupling strength of micro-comb fingers with electrostatic load is stronger than that without electrostatic load.

### 4.3. Micro-Comb Finger Thermoelastic Behaviors for Bending Vibration in Sense-Mode 

**Figure 6:**
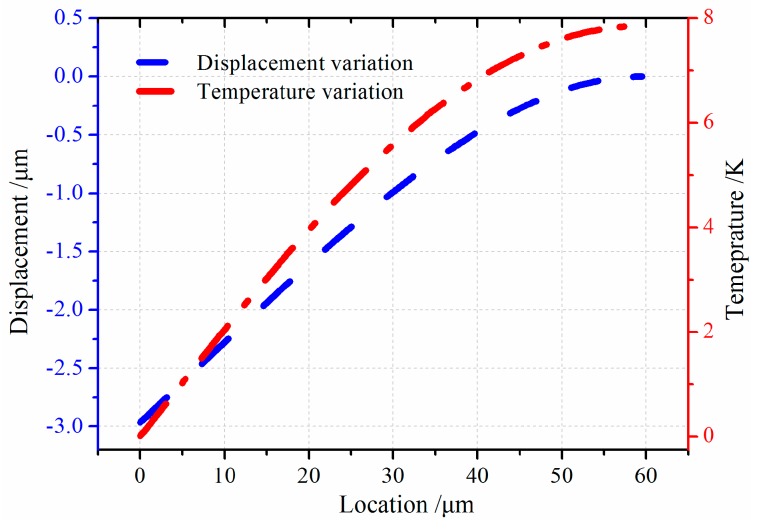
Field quantities variation under transverse vibration.

When the gyroscope vibrates at the *x* axial then the resonator will vibrate at the *y* axial as rotating force is applied to the *z* axial. The *x* axial is defined as drive-mode and *y* axial is called sense-mode. The resonator performs longitudinal vibration at drive-mode and flexural vibration at sense-mode. Then the thermoelastic behaviors of the resonator should be considered in terms of transverse vibration. Figure 6 shows displacement and temperature variation of resonator bending vibration. Temperature has the opposite variation with displacement. The resonator deflection shares zero at the fixed end while the temperature has the maximum value. It is attributed to the presence of maximum stress of the resonator’s fixed end, which results in the peak temperature at this point. At the free end is also a similar trend.

### 4.4. Simulation Results

**Figure 7:**
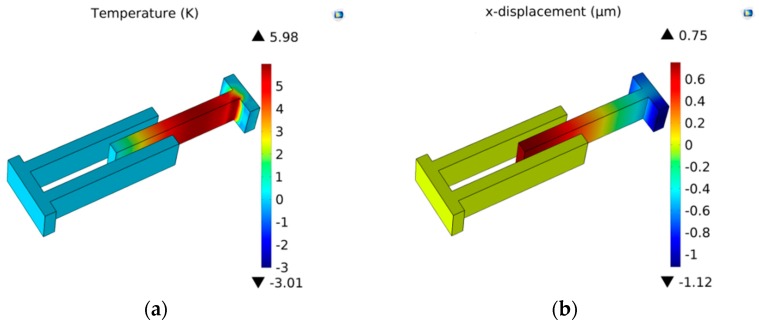
Distribution of temperature and variation of displacement for electrostatically actuated longitudinal vibration: (**a**) temperature displacement; (**b**) displacement variation.

**Figure 8:**
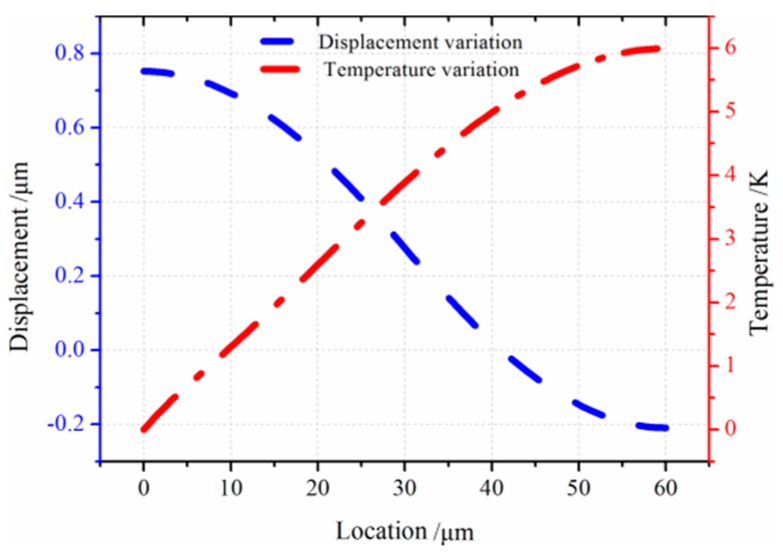
The path analysis result of temperature distribution and displacement variation.

**Figure 9:**
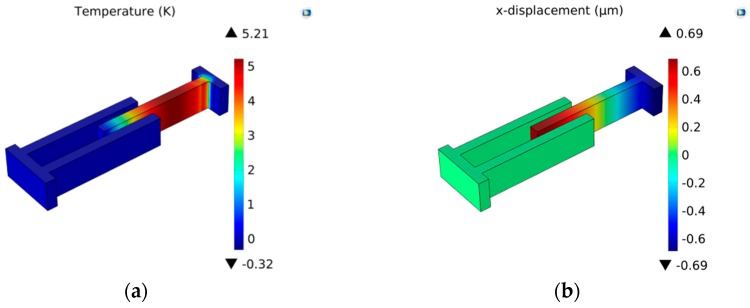
The temperature variation and displacement distributionfor longitudinal vibration without electrostatic load: (**a**) temperature variation; (**b**) displacement distribution.

**Figure 10:**
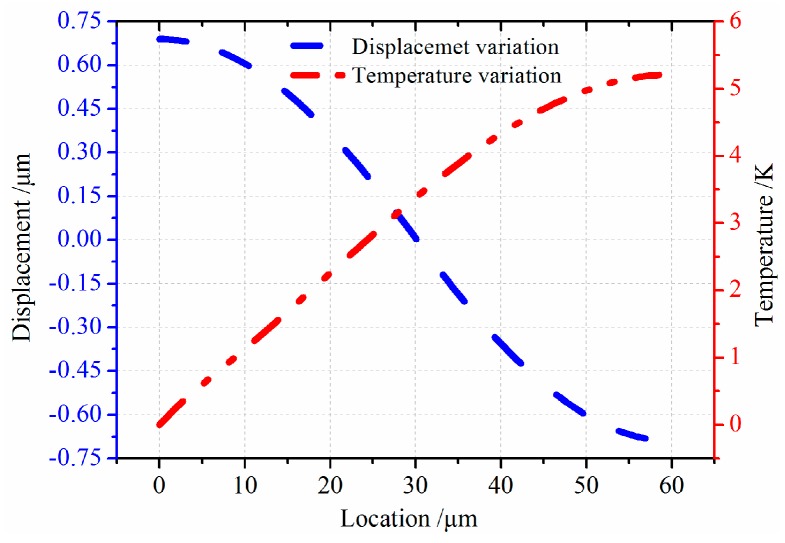
The path analysis results of temperature and displacement variation.

**Figure 11:**
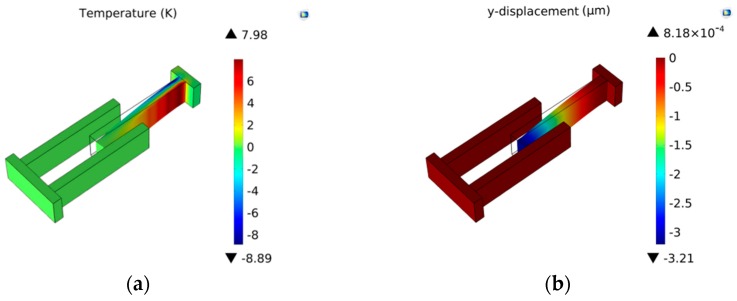
The temperature variation and displacement distribution for transverse vibration: (**a**) temperature variation; (**b**) displacement distribution.

**Figure 12:**
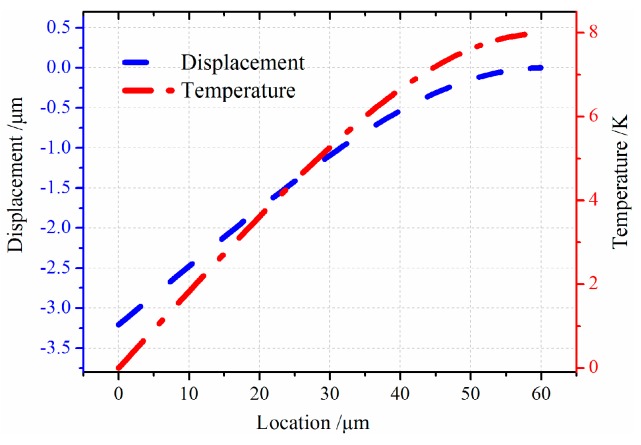
The path analysis results of temperature and displacement variation.

In this section, models of the micro-comb finger in the presence of TED are investigated with COMSOL Multiphysics software. A 3-D computational model that provides the approximation of TED for both vibrations at drive-mode and sense-mode is developed. The geometry dimension is illustrated in Table 1. The mesh generation is implemented by adopting the mapping approach. To eliminate the effect of air on the resonator, the simulation is carried out under vacuum (below 0.01 mTorr). The initial and mechanical boundary conditions are based on the former setting (Equation (8)). The forced vibration characteristics at the first longitudinal mode are shown in Figure 7. The path analysis is made through selecting a line along the *x* direction in the movable comb upper face. The results are presented in Figure 8. It is observed that the longitudinal vibration *u* is symmetrically distributed at the range of −0.752~0.752 µm and the temperature *θ* increases directly with the location at the resonator length direction changing. Figure 9 provides the simulation results of vibration without electrostatic load. The contour plots of the temperature distribution and the vibration deformation are presented. From the figures, the maximum value of generated temperature is 5.210 K which is smaller than that with electrostatic-actuated vibration. Moreover, the displacement maximum value is smaller than that with forced vibration. The same analysis is applied in Figure 9, and the results are shown in Figure 10. Compared with the electrostatic-actuated vibration, the temperature and displacement distribution follow an similar pattern except for the extreme value. The sense-mode vibration characteristics are shown in Figure 11, and the path analysis of numerical results is illustrated in Figure 12. The field quantities of drive-mode and sense-mode are presented in Table 2, illustrating that the theoretical values coincide with simulation results within some small errors. Moreover, from Table 2 it can be seen that thermoelastic behaviors at sense-mode are more sensitive than those that at drive-mode. 

## 5. The Thermoelastic Damping and Quality Factor

In this section, the parameter of TED that characterizes the energy dissipation at the vibration process is discussed. The thermal energy method will be used to calculate the thermoelastic damping. Landau and Lifshitz derived expressions for energy dissipation due to thermoelastic coupling effect by seeking the dissipated vibration energy, which is equal to the amount of heat flowing from a hot to cold region [21]. The assumption is made that the lost energy per cycle of vibration is all transformed into thermal energy. The dissipated energy is thus presented as:

(32) $$\Delta W={\int_{0}^{2\pi /\omega }\int_{V}\frac{\kappa }{T_{0}}\left(\nabla \theta \right)}^{2}dVdt$$

The stored mechanical energy per cycle of vibration is:

(33) $$W=\frac{1}{2}\int_{V}\left(\sigma \varepsilon \right)dV$$

**Figure 13:**
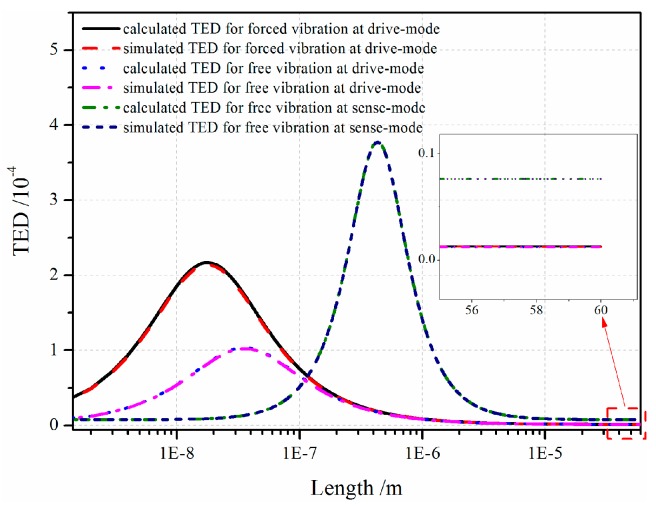
TED under different conditions.

From the above graphics, it can be found that the dissipated energy and stored mechanical energy per cycle have been deduced. The TED is defined as below.

(34) $$Q_{\mathrm{TED}}=\Delta W/2\pi W={\int_{0}^{2\pi /\omega }\int_{V}\frac{\kappa }{T_{0}}\left(\nabla \theta \right)}^{2}dVdt/\pi \int_{V}\left(\sigma \varepsilon \right)dV$$

where σ and ε are the stress and strain under vibration process. On account of inverse Laplace transformation of *s* domain to time domain, the dependence of TED on resonator material and geometry dimension is deduced. It is difficult to find the inverse Laplace transformation in the Laplace domain analytically, so the numerical results for different conditions are plotted in Figure 13. The results show that as the resonator length increases, the TED first increases and then decreases. Furthermore, there exists a critical size at which TED takes the maximum value. It is shown that under different vibration modes, the TED reaches different peak values. TED for electrostatic-actuated vibration at drive-mode is larger than that for free vibration, and TED of resonator vibration at sense-mode is larger than that at drive-mode. For the movable comb resonator with 60 µm length, TED at drive-mode of electrostatic-forced vibration and free vibration and sense-mode vibration is 1.278 × 10^−6^, 1.212 × 10^−6^, 7.662 × 10^−6^ and the maximum TED value with critical size is 1.9 × 10^−8^ m, 3.6 × 10^−8^ m, 4.3 × 10^−7^ m, respectively. 

Once TED is known, the quality factor related to the TED in the tuning-fork resonator can be calculated as

(35) $$Q=Q_{TED}^{-1}=2\pi W/\Delta W$$

**Figure 14:**
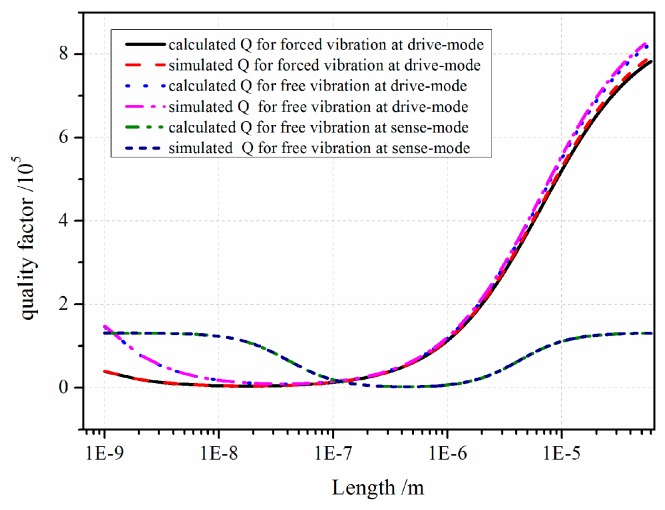
Quality factor under different conditions.

The numerical results of *Q* are shown in Figure 14. It shows that with the vibration strength increasing, the dissipated thermal energy also increased. Therefore, *Q* of the movable resonator for vibration at sense-mode shares the minimum value. For an intuitive impression, the numerical result *Q* within theoretical and simulation methods is listed in Table 3.

Table 3 clearly shows that theoretical values are in line with simulation results within a small fluctuation, verifying the theoretical model. We conclude that within vibration at sense-mode, more mechanical energy is dissipated through thermal energy, and its quality factor is correspondingly lower than that at drive-mode. 

## 6. Experimental Verification

To verify the thermoelastic coupling effect on the tuning-fork resonator, the experimental conclusions are used from the paper of Xu [22]. In Xu’s results, the model consists of a tuning-fork comprised of a set of three flexural beams and two proof masses, and is fixed on the substrate through the two anchors located at the center of the whole device. The study in this thesis focuses on one group comb finger. According to the experimental conclusions from Xu, the quality factor expression can be written as

(36) $$Q_{mesured}=\frac{\omega L_{io}}{R_{io}+R_{0}}$$

where $R_{o}$ represents the external resistor.$L_{io}$, $R_{io}$ is the equivalent circuit model of the tuning-fork structure consisting of a RLC circuit in series, and the values of the equivalent electrical components of the structure can be calculated as follows.

$$L_{io}=\frac{M}{V_{dc}^{2}{\left(dC/dx\right)}^{2}},\;R_{io}=\frac{\sqrt{k\cdot M}}{V_{dc}^{2}{\left(dC/dx\right)}^{2}Q}$$

where *M* and *k* are the modal mass and stiffness, respectively.

From Xu’s results, the TED for a whole device is the dominant loss in the drive-mode and sense-mode of the tuning-fork structure, occupying as high as 76% of the total energy loss. The comb finger parameters are substituted to the *Q* expression in Xu’s experimental research, which can obtain the relationship between *Q* and resonator length. The comparison was made between the simulated results of comb finger and the calculated results from Equation (36).

**Figure 15:**
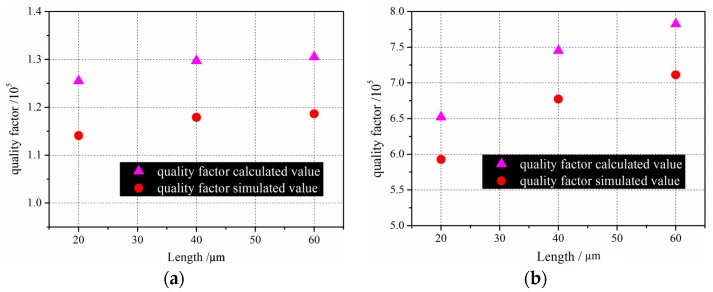
The compared *Q* values of the drive-mode and sense-mode of micro-comb finger versus different lengths (20 µm, 40 µm, 60 µm). (**a**) drive-mode and (**b**) sense-mode.

From the comparison results in Figure 15, the simulated *Q* values are about 10% smaller than the results from Equation (36). According to Xu, the sum of electronics damping and anchor loss is about 20% of measured *Q*. The electronics damping is not considered in the simulation process, so the simulated *Q* value is a little smaller than the calculated value. The analytical solution coincides with the calculated results from Equation (36). The analytical conclusions are thus experimentally verified.

## 7. Conclusions

Aimed at the internal dissipation due to thermoelastic coupling in tuning-fork resonator, this paper established the thermoelastic coupling governing equations for vibrations at drive-mode and sense-mode. The numerical results of vibration displacement and the temperature which generated in vibration process are then calculated, and the comparison is made between numerical results and simulation results. From that, the calculated results of displacement and temperature coincide with the FEM simulated results. Through the results, the generated heat due to the vibration process shares a small magnitude. In other words, the loss energy enjoys a lower level compared with the vibration energy. The temperature generated under three conditions is 5.943 K, 4.950 K and 5.210 K, respectively. Also, the error between theoretical and simulated results is quite small. Additionally, TED and *Q* are analyzed with theory and simulation. By means of other researchers’ experimental conclusions, the analytical and simulated results can be verified. The TED numerical results have the order of 10^−6^ for 60 µm length micro-comb finger at both drive-mode and sense-mode vibrations. At the drive-mode vibration, the electrostatically actuated TED is slightly higher than that at the free vibration. For the transverse vibration, the calculated value is 7.662 × 10^−6^, which is larger than the drive-mode vibration. The comparison is thus made between the simulated *Q* value and the calculated *Q* value based on Xu’s conclusion, thereby showing that the TED is the dominant loss for a gyroscope with tuning-fork resonator in the drive-mode and sense-mode. 

## Figures and Tables

**Table 1:** Mechanical, thermal, physical and electrical properties of gyroscope with silicon material.

**Mechanical**	E/GPa	υ	$\rho /\mathrm{kg}\cdot m^{-3}$		**Thermal**	${\alpha /K}^{-1}$	$\kappa /W\cdot m^{-1}\cdot K^{-1}$	$C_{v}/J\cdot {\mathrm{kg}}^{-1}\cdot K^{-1}$	$T_{0}/K$
Values	165	0.22	2330		values	2.6 × 10^−6^	159	713	300
**Physical**	L/m	h/m	b/m	g/m	**Electrical**	$V_{\mathrm{ac}}/V$	$V_{\mathrm{dc}}/V$	$\varepsilon /F\cdot m^{-1}$	t/μs
Values	60 × 10^−6^	15 × 10^−6^	5 × 10^−6^	5 × 10^−6^	Values	3	15	8.854 × 10^−12^	0.01

**Table 2:** Field quantities simulation and analytical comparison results for vibration at drive-mode and sense-mode.

Vibration Mode			Displacement/µm		Temperature/K	
Drive-mode	Forced vibration	Theoretical value	0.735	−0.735	5.943	0
		Simulation value	0.752	−0.752	5.977	0
		Error rate (%)	2.312	2.312	0.572	/
	Free vibration	Theoretical value	0.667	−0.698	4.950	0
		Simulation value	0.69	−0.750	5.210	0
		Error rate (%)	3.448	3.448	5.252	/
Sense-mode	Theoretical value		−2.966	0	0	7.85
	Simulation value		−3.17	0	0	7.98
	Error rate (%)		6.878	/	/	1.656

**Table 3:** TED and *Q* simulation and analytical results for vibration at drive-mode and sense-mode.

Vibration Mode	Drive-Mode						Sense-Mode		
	Forced Vibration			Free Vibration					
	Theoretical Value	Simulation Value	Error Rate %	Theoretical Value	Simulation Value	Error Rate %	Theoretical Value	Simulation Value	Error Rate %
Frequency/HZ	4.406 × 10^8^	4.409 × 10^8^	0.068	4.403 × 10^8^	4.406 × 10^8^	0.068	2.264 × 10^8^	2.261 × 10^8^	0.133
TED	1.278 × 10^−6^	1.260 × 10^−6^	1.41	1.212 × 10^−6^	1.198 × 10^−6^	1.259	7.662 × 10^−6^	7.746 × 10^−6^	1.10
*Q*	7.825 × 10^5^	7.937 × 10^5^	1.47	8.251 × 10^5^	8.347 × 10^5^	1.163	1.305 × 10^5^	1.291 × 10^5^	1.07
